# Comparative Study of Anatomical and Learned Features in AI Models for Structural Brain MRI

**DOI:** 10.21203/rs.3.rs-10851563/v1

**Published:** 2026-09-09

**Authors:** Boyang Yu, Miquel Lopez Escoriza, Long Chen, Arjun V. Masurkar, Narges Razavian, Carlos Fernandez-Granda

**Affiliations:** 1 Center for Data Science, New York University, NYU, 60 Fifth Ave, New York, 10011, New York, United States.; 2 Department of Neurology, NYU Grossman School of Medicine, 550 1st Ave, New York, 10016, New York, United States.; 3 Courant Institute of Mathematical Sciences, NYU, 251 Mercer St, New York, 10012, New York, United States.

**Keywords:** Deep learning, pretraining, neuroimaging, brain anatomy, segmentation

## Abstract

In this work, we comprehensively evaluate three popular feature-extraction paradigms in AI-based neuroimaging modeling: (1) computation of anatomical surfaces and volumes, (2) supervised learning with convolutional neural networks (CNNs), and (3) unsupervised pretraining of vision transformer (ViT) foundation models, followed by supervised finetuning. Our study is based on 18 publicly available datasets containing 3D structural T1-weighted MRI scans from approximately 80,000 participants across seven distinct clinical tasks. We observe that a linear model based on anatomical features matches the diagnostic performance of complex nonlinear features learned by sophisticated AI frameworks, including foundation models trained on thousands of scans. Conversely, CNNs and pretrained ViTs learn features that implicitly capture relevant anatomical information, bypassing the need for explicit feature extraction. Building upon these insights, we propose Anatomy Segmentation Pretraining (ASP), a novel method to incorporate anatomical information during foundation-model pretraining, which outperforms existing models in biological age estimation.

## Introduction

Advances in machine learning and computer vision have exerted a major influence on neuroimaging over the past two decades. Initially, these models relied on hand-engineered features, such as hippocampal volume or cortical thickness, combined with classical statistical models (e.g. logistic regression or support vector machines) [1-4]. Subsequently, modern convolutional neural networks (CNNs) enabled automatic learning of complex features, which are challenging to interpret anatomically, but achieved strong performance for a variety of neuroimaging tasks [5-8]. More recently, foundation models based on vision transformers (ViTs) [9] are able to learn features from vast unlabeled datasets [10, 11]. They can then be finetuned for different downstream tasks. Several recent works adapt this approach to neuroimaging data [12-15], for example to develop multimodal models.

In this work, we perform a comparative study of AI models for neuroimaging, with a focus on 3D structural T1-weighted MRI scans. T1-weighted MRI is a standard imaging modality that enables visualization of anatomical detail at a high resolution and is a routine part of most clinical protocols. Figure 1 provides an overview of the study. We consider seven different clinical tasks: diagnostics of Alzheimer’s disease, epilepsy, Parkinson’s disease, identification of tumor IDH mutations, brain age prediction and segmentation of brain tumors and stroke lesions. Overall, we use 18 different publicly-available datasets with more than 96,000 MRI scans from approximately 80,000 participants for training and evaluation.

Our overarching goal is to compare the three main strategies to obtain features for AI-based modeling in neuroimaging: (1) extraction of preselected anatomical characteristics such as volumes and surfaces of brain regions, (2) supervised learning with convolutional neural networks, and (3) unsupervised pretraining of foundation vision transformers on large general-population cohorts, followed by supervised finetuning. As reported in the Results section, the three strategies yield similar performance across the different tasks. Our results indicate that preselected anatomical features match the diagnostic performance of complex nonlinear features learned by sophisticated AI frameworks, including foundation models trained on thousands of scans. Conversely, convolutional neural networks and pretrained vision transformers learn features that implicitly capture relevant anatomical information, bypassing the need for computationally intensive feature extraction.

In the case of ViTs pretrained on a large general-population cohort, we show that anatomical information is learned already during the pretraining stage, when the learned features become increasingly predictive of different brain structures. Inspired by this observation, we propose a novel pretraining strategy (Anatomy Segmentation Pretraining or ASP), which aims to incorporate explicit anatomical grounding in the training of neuroimaging foundation models. As described in the Methods section, this is achieved by forcing the model to perform segmentation of relevant brain structures during pretraining. While this approach performs on par with the other strategies across the clinical tasks, it achieves superior performance in estimating biological age, a task for which a larger number of training data is available.

In summary, this comparative study demonstrates that explicit anatomical features remain competitive with representations learned by advanced deep learning models, particularly across clinical cohorts ranging from dozens to thousands of training examples. Furthermore, we introduce a novel, anatomy-guided pretraining framework that explicitly leverages anatomical information to enhance the pretraining of neuroimaging foundation models.

## Results

Figure 1 provides an overview of the study. We compare the following AI models, adapted to process 3D MRI scans, across different tasks. The models are chosen to represent the main current approaches for image classification and segmentation in data-driven computer vision.

- Linear logistic-regression models based on anatomical features consisting of 178 volumes and surfaces of brain regions (denoted Anatomical in the figures).
- Convolutional neural networks, which are either ResNets [16] for classification tasks (denoted CNN) or U-Nets [17] for segmentation tasks.
- Vision-transformer models [9] pretrained on a large pretraining cohort via masked autoencoding (denoted Pretrained ViT).
- ViTs pretrained using a novel strategy (Anatomy Segmentation Pretraining, denoted ASP ViT) that leverages anatomical information extracted via segmentation from the scans in the pretraining cohort.

The pretraining cohort includes 69,878 MRI scans from 65,249 subjects, primarily from UK Biobank[18]. More details about the pretraining data are provided in the appendix (Section A.2). In addition, we also include comparisons with BrainIAC [14], a ViT pretrained using contrastive learning on a different pretraining cohort of 32,000 MRI scans, and UNETR [12], a medical image segmentation architecture that combines a ViT encoder with a U-Net–style decoder.

The models are trained to perform the following seven tasks. The results are shown in Figure 2. Additional details about the datasets used to build the training and evaluation cohorts for each task, and the pretraining cohort, are provided in Table 1 and in the appendix (Section A).

- **Diagnostics of Alzheimer’s disease:** The goal is to distinguish between healthy controls, patients with mild cognitive impairment and patients with Alzheimer’s disease. The training cohort consists of n=1,453 subjects from ADNI [19]. Evaluation was carried out on an internal test cohort extracted from ADNI (n=364), and seven external cohorts, extracted from NACC [20] (n=4,926), AIBL [21] (n=664), MIRIAD [22] (n=69), and OASIS-1 (n=416), OASIS-2 (n=150), OASIS-3 (n=1,044) and OASIS-4 (n=180) [23-25]. For the internal test cohort and six external cohorts, ASP ViT has the highest macro-AUC, but the difference with respect to the CNN and the anatomical logistic regression model is only statistically significant for OASIS 2. We didn’t apply BrainIAC to this task because its pretraining cohort includes several of our external test cohorts.
- **Diagnostics of Parkinson’s disease:** The goal is to distinguish between patients with Parkinson’s and healthy controls. A training cohort (n=1,572) and an internal test cohort (n=460) were extracted from PPMI [26]. The Pretrained ViT achieves the highest AUC, but the difference is only statistically significant with respect to BrainIAC.
- **Detection of tumor IDH mutations:** The goal is to identify brain tumors with Isocitrate Dehydrogenase (IDH) mutation. A training cohort (n=1,874) and an internal test cohort (n=106) were extracted from UCSF-PDGM [27], which contains labels obtained via genetic sequencing of tissue acquired during biopsy or resection. ASP ViT achieves the highest AUC, but the difference with the remaining models is not statistically significant.
- **Diagnostics of epilepsy:** The goal is to distinguish between patients with epilepsy and healthy controls. A training cohort (n=82) and an internal test cohort (n=81) were extracted from OpenNeuro [28]. The models were also applied to Episurg [29], an independent cohort containing only epilepsy cases (n=268). The Pretrained ViT achieves the highest AUC, but the difference with the remaining models is not statistically significant. We could not apply the CNN to this task due to the limited training data.
- **Whole tumor segmentation:** The goal is delineation of adult brain gliomas. A training cohort (n=1,000) and an internal test cohort (n=251) were extracted from the BraTS 2023 Adult Glioma challenge [30-32]. The U-Net achieved the highest Dice score (slightly above ASP ViT), but the difference is only statistically significant with respect to UNETR. If the training data is reduced to 10%, ASP and Pretrained ViT outperform U-Net and UNETR.
- **Segmentation of stroke lesions:** The goal is segmentation of brain lesions associated with stroke. A training cohort (n=355) and an internal test cohort (n=300) were extracted from ATLAS R2.0 [33]. The Pretrained ViT achieved the highest Dice score (slightly above ASP ViT), but the difference is only statistically significant with respect to UNETR.

Overall, we observed that there was no significant difference between the pretrained ViTs, the CNNs and linear models based on anatomical features across the different tasks.

We conjecture that the inability of the more complex deep-learning models to outperform the simpler linear model is due to the limited training data available for each task. In order to investigate this further, we trained the models to estimate biological age using a much larger training cohort drawn from UK Biobank, HCP, CamCAN and IXI (n=62,890). Evaluation was carried out on a held-out subcohort from UK Biobank (n=6,950), and on two external cohorts of healthy subjects extracted from ADNI (n=2,791) and NACC (n=1,136).

Figure 3 shows the results on age estimation. The ASP ViT model outperforms the rest, achieving a mean absolute error (MAE) of 2.54 years in the internal test cohort (a) and of 4.19 years in the NACC test cohort (b) and the ADNI test cohort (c). The Pretrained ViT is close in performance on the internal test cohort (0.35 years greater), but the gap widens for the external cohorts (more than 1 year greater). The logistic regression model performs worse than the ViT approaches by a significant margin (between 1 and 2 years), but clearly outperforms the CNN. The performance differences are maintained when stratifying the data by sex. Stratifying by age reveals that the differences between the models are mainly driven by the superior performance of the ViT models (particularly ASP ViT) on subjects with advanced ages (above 80), which are less represented in the training and pretraining cohorts, especially with respect to the CNN.

The best-performing age-estimation model was also applied to mildly-cognitively impaired (MCI) and dementia patients from the NACC and ADNI datasets, as shown in Figure 3(b,c). Age estimates for dementia patients were consistently above their biological age, in most age intervals for both cohorts. The effect also exists to some extent for MCI subjects, but is less pronounced.

## Methods

In this section, we first present a new technique for pretraining vision transformers (ViTs) using anatomical information. Then, we describe the remaining methodology in our study, including masked autoencoder pretraining of ViTs, downstream application of pretrained ViTs, linear models based on anatomical features, convolutional neural networks, and data preprocessing.

### Anatomy Segmentation Pretraining of Vision Transformer

We propose a novel approach to pretrain ViTs on MRI data. The goal is to inject anatomical information into the model during pretraining. The motivation is that simple models that rely only on anatomical information, such as the logistic-regression model with brain-region volume and surface features used in this study, can be very effective (see Results section).

We build upon a key observation: when pretraining ViTs via masked autoencoding (MAE), the model automatically learns embeddings that reflect anatomical information, but only to some extent. The green region in the graph from Figure 4(b) shows the accuracy of predicting the volumes of different brain regions from MAE embeddings via linear probing during training. The accuracy increases, indicating that the embeddings gradually capture anatomical information. In order to further boost the anatomical information learned by the model, we propose to incorporate brain-region segmentation as an auxiliary task during pretraining.

Our procedure to perform anatomy segmentation pretraining (ASP) is illustrated in Figure 4(a). A 3D ViT produces the embedding used for downstream tasks. After MAE pretraining (green region in Figure 4(b)), we train a lightweight mask decoder to perform segmentation of different brain regions in two phases. First, the ViT parameters are frozen to ensure that the decoder learns how to use the existing embedding for segmentation (yellow region in Figure 4(b)). Next, the encoder and decoder are trained jointly. In this ASP phase, segmentation and MAE pretraining are performed in alternating epochs to prevent the model from *forgetting* useful features learned via MAE. This strategy is common in continual-learning methods that balance new-task adaptation with the retention of prior knowledge [34-36].

Regions for ASP were selected based on their disease relevance and anatomical scale. We discarded regions that lacked a strong disease association and contributed less than 1% of the total brain volume. To further improve efficiency, symmetrical structures (such as the left and right hippocampus) and anatomically related sub-units (such as segments of the corpus callosum) were consolidated into single labels. This resulted in 39 distinct regions, listed in Figure 4(c). More details are provided in the Appendix Section A.7.

The segmentation architecture used to perform ASP is tailored specifically to the pretraining task. Deep-learning segmentation models typically rely heavily on skip connections, which emphasize local feature fusion and reduce the importance of global representations [17], [12]. In contrast, the goal of ASP is to concentrate the anatomical information in the ViT embedding. To address this, we use a bottleneck decoder architecture(see Figure C1), where the segmentation prediction is generated directly from the ViT embedding, without any skip connections. The decoder is lightweight, in order to further encourage the model to concentrate rich anatomical information in the ViT transformer. More details are provided in the Appendix Section C.2.

A key challenge is applying ASP to a relatively large number of anatomical regions (39, as explained above). A naive implementation based on a standard softmax output would be too memory intensive, as it would require generating 39 different 3D logit maps (39×128×128×128). Instead, we adopt a prompt-based architecture, inspired by [37], where a prompt is used to adapt the Segment Anything Model [38] to different segmentation tasks. A *brain-region prompt* indicates which brain region should be segmented by the lightweight decoder, and the decoder output is compared to a binary segmentation task indicating the appropriate region (see Figure 4(a)). The brain region is selected at random during pretraining.

We trained the ASP model for 400 epochs with a batch size of 16 per GPU. We used binary cross entropy and the AdamW optimizer (*β*_1_ = 0.9, *β*_2_ = 0.95, weight decay = 0.04), with a base learning rate of 5 × 10^−4^. We applied learning rate scheduling via cosine annealing with linear warm-up over the first 5 epochs. Convergence is observed at around 200 epochs, requiring approximately one month of training on a single 42GB NVIDIA RTX8000 GPU.

Figure 4(c) reports the performance of the decoder for the different regions before and after the ASP phase, showcasing that the model is indeed able to learn how to segment the different regions reasonably accurately (although its performance for smaller or more intricate region is relatively modest). Figure 4(d) shows some examples of segmentation outputs, compared to the ground truth. Figure 4(e) provides an ablation study, which establishes that each element in the proposed framework (prior MAE pretraining, initial freezing of the encoder to train the decoder, and alternating with MAE to avoid forgetting) all provide a non-negligible increase in performance for the downstream task of age estimation. Further information about the ablation study is provided in C.3.

### Masked Autoencoder Pretraining of ViTs

Masked Autoencoding (MAE) is a self-supervised learning framework that trains ViTs by reconstructing masked images, which are missing a random subset of their pixels [11]. We adapted this framework to process 3D structural MRI volumes. We used a ViT-Base encoder adapted to 3D inputs, consisting of 12 transformer blocks with 12 attention heads and a 768-dimensional embedding, using 16×16×16-voxel patches. During pretraining, 75% of the 3D patches in each volume were randomly masked, and the ViT was trained to reconstruct the original volumes from the unmasked patches. Volumes were represented as single-channel inputs with intensities scaled to [0, 1]; images were padded to 256 × 256 × 256, randomly cropped to 128 × 128 × 128, and augmented with random flipping and intensity shifting. Pretraining used mean squared error (MSE) reconstruction loss for 1000 epochs with batch size 32 per GPU, AdamW [39] (*β*_1_ = 0.9, *β*_2_ = 0.95, weight decay = 0.005), and a cosine learning rate schedule [40] with base learning rate 0.0015 and linear warm-up during the first 2.5% of training steps. Convergence is observed at around 900 epochs, which took around 10 days on two 80GB NVIDIA A100 GPUs.

### Downstream Application of Pretrained ViTs

To adapt the pretrained ViTs for downstream applications, we feed the resulting embeddings into task-specific output modules: a binary-classification head for diagnostic tasks, a linear-regression head for regression tasks (e.g. estimation of age or of anatomical features) and a U-Net–style decoder for voxel-level segmentation, following the UNETR architecture [12]. See Section C.1 for additional details. The pretrained ViTs were adapted to the different downstream tasks using the following strategies.

- **Linear probing:** The linear regression and classification heads were trained to generate predictions from the ViT embedding, using the sklearn implementations of logistic regression and ordinary least squares. This approach was used as a diagnostic tool during pretraining to monitor representation quality using anatomical and demographic targets, as illustrated by Figure 4(b). It was also used for diagnostics of Parkinson’s disease and detection of tumor IDH Mutation, due to the small size of the training datasets.
- **Low-Rank Adaptation (LoRA):** Both ViT encoders (ASP and pretrained) were adapted using LoRA [41, 42]: the original model parameters were kept frozen, and trainable low-rank matrices were introduced into the attention layers. These low-rank parameters were optimized jointly with the task-specific heads, substantially reducing the number of trainable parameters compared with updating the entire encoder. Random cropping was employed for regularization during training. At inference time, we used center cropping for classification tasks and a sliding window approach for full-volume segmentation tasks. Training was performed with the AdamW optimizer for 150 epochs.
- **Full Finetuning:** For ablation experiments, we updated all parameters from the ASP ViT encoder jointly with the downstream head using the same optimization and augmentation settings as LoRA. See Appendix Section C.

### Linear Models with Anatomical Features

For each of our downstream diagnostic tasks, as well as age estimation, we trained linear models based on 178 anatomical features extracted via FreeSurfer [43] (v7.4.1), using the recon-all pipeline. The features include regional volume measurements, cortical surface areas, and cortical thickness values. A complete list is provided in Appendix Table A3. The resulting models are referred to as *Anatomical* in the Results section. These linear models achieved similar performance as nonlinear alternatives; details are provided in Appendix Section B.1. For Alzheimer’s diagnosis, we encoded healthy as 0, Alzheimer’s as 1 and mild cognitive impairment as 0.5, as this produced better results than treating diagnosis as a multiclass classification task [44], see Appendix Section B.2.

### Convolutional Neural Networks

We used the ResNet-18 architecture [16] for diagnostic classification tasks and the U-Net architecture [17] for segmentation tasks, based on preliminary experiments. For classification, the models were trained for up to 60 epochs using a batch size of 16, stochastic gradient descent (SGD) [45], an initial learning rate of 0.01, and early stopping based on validation loss. For Alzheimer’s diagnosis, we used the same label encoding as in the logistic regression model, and optimized a binary cross-entropy objective during training. Additional analyses on baseline CNN selection are reported in Appendix Sections B.3.

### Data Preprocessing

To harmonize data across cohorts, we implemented a standardized preprocessing pipeline for all MRIs. While datasets for segmentation tasks (BraTS and ATLAS R2.0) were already standardized, all other raw data—initially in DICOM or NIFTI format—underwent a uniform processing sequence. DICOM series were first converted to NIFTI using the dcm2nii tool.

Except for the pre-standardized segmentation cohorts, scans were processed using FreeSurfer’s recon-all workflow, which consists of three sequential phases (initial processing, surface reconstruction and refinement). This workflow also served as our primary quality control mechanism; any images that failed the initial processing phase were excluded from the study. A detailed description of the recon-all execution time and workflow is provided in Appendix Section A.5.

Finally, to ensure spatial alignment, all images and tissue masks were coregistered to the MNI152 2009a Nonlinear Symmetric template using the mri vol2vol command. The resulting preprocessed scans have a standardized volume of 197 × 233 × 189 voxels with an isotropic resolution of one cubic millimeter.

### Statistical analysis

In each experiment, we report the overall metric and 95% confidence interval (CI), calculated by bootstrapping the held-out test set 1000 times. Specifically, we generate 1000 bootstrap samples by resampling the test set with replacement, compute the metric on each sample, and estimate the 95% CI from the 2.5th and 97.5th percentiles of the resulting metric distribution.

### Ethics Approval and Consent to Participate

This study used de-identified, secondary data from publicly available neuroimaging repositories (UKBB, HCP, CamCAN, IXI, NACC, ADNI, OASIS, AIBL, MIRIAD, PPMI, OpenNeuro, EPISURG, UCSF-PDGM, BraTS, ATLAS). Each dataset was collected under institutional ethics approval and informed consent obtained by the original data-collecting institutions, as described in their respective publications. No new human data were collected for this study.

## Discussion

Our study demonstrates that across a wide range of publicly available structural MRI datasets, linear models trained on preselected anatomical features remain highly competitive with deep neural networks. While traditional segmentation approaches are often computationally expensive for feature extraction, modern deep learningbased segmentation frameworks (e.g., FastSurfer [46]) offer an efficient alternative. Our results suggest that leveraging these deep-learning tools to extract structured anatomical features for linear models provides a computationally efficient, interpretable, and robust baseline for various diagnostic tasks.

Furthermore, our results indicate that diagnostic performance using structural MRI may have reached a saturation point within existing public datasets that contain only hundreds to a few thousand scans. Further progress will likely require the curation of substantially larger cohorts. Our age-estimation experiments on a cohort of 60,000 subjects indicate that scaling public datasets could unlock further methodological innovation.

From the methodological perspective, we show that vision transformers learn relevant anatomical structure during unsupervised pretraining, but at a slow rate that can be greatly accelerated using anatomical segmentation as an auxiliary pretraining task. This suggests that leveraging clinically-relevant tasks could mitigate the small size of medical pretraining datasets when compared to general computer-vision pretraining databases with millions of examples.

## Supplementary Material

Supplement 1

## Figures and Tables

**Fig. 1:**
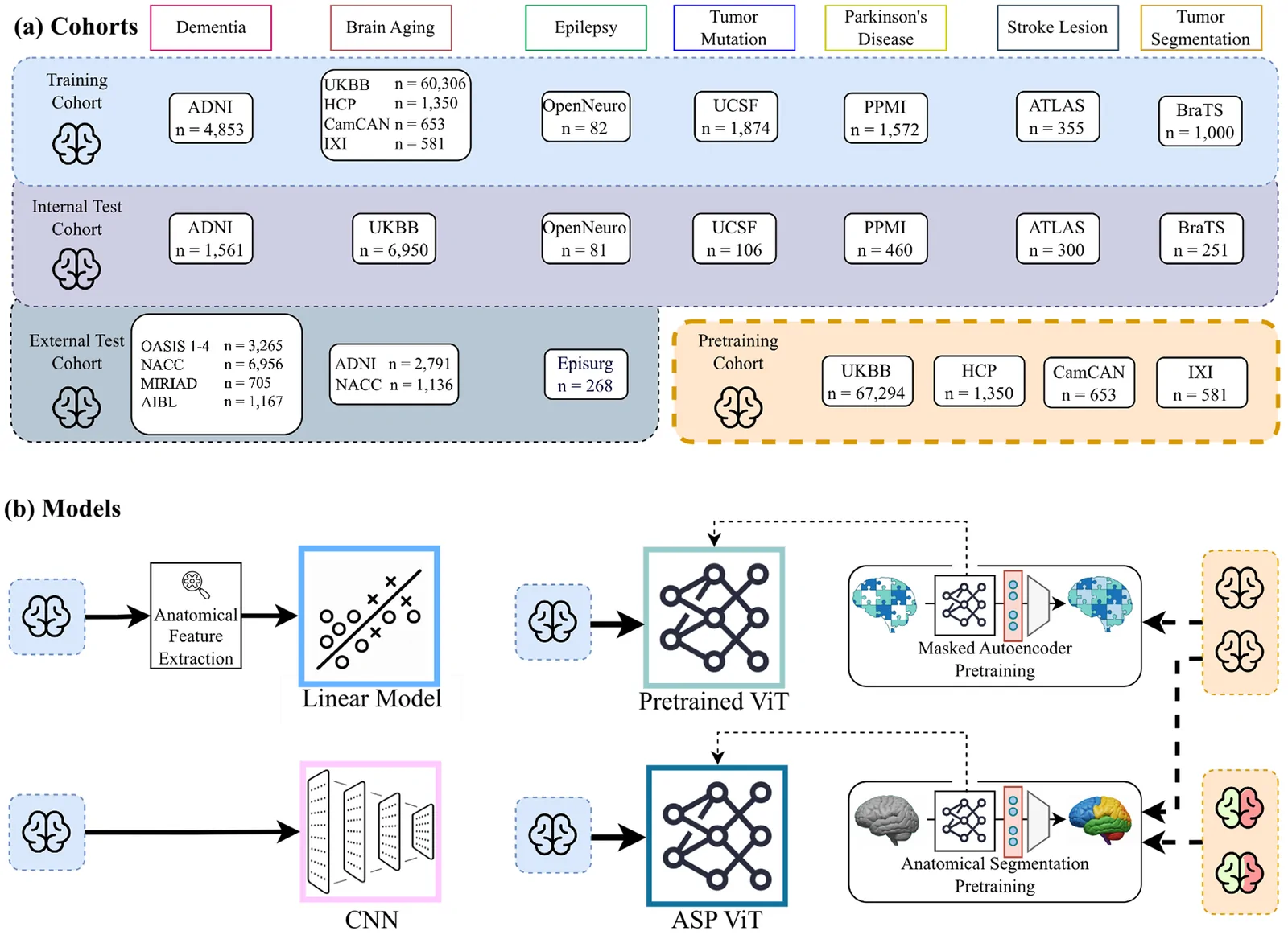
Overview of the study. **(a) Cohorts used in the study.** Cohorts used for pretraining, downstream training, internal testing, and external evaluation. Reported *n* values denote MRI scans; all splits were performed at the participant level, such that all scans from the same participant were assigned to the same split. External cohorts were drawn from independent data sources. **(b) AI models included in the study**. These represent the main feature-extraction approaches for neuroimaging data: linear models based on anatomical features, convolutional neural networks (CNNs) and vision transformers (ViTs) pretrained on large general-population cohort. For the ViTs, in addition to standard pretraining based on masked autoencoding, we propose a novel scheme called Anatomical Segmentation Pretraining (ASP).

**Table 1:** Datasets used to build the pretraining cohort, and the training and evaluation cohorts for each task in the study.

Dataset	Number of scans	Number of subjects	Age (years) (mean ± std)	Gender (female %)	Brain volume in mL (mean ± std)
UKBB	67294	62665	64.93 ± 7.69	52.81%	1156 ± 114
HCP	1350	1350	38.66 ± 25.76	54.97%	1179 ± 137
CamCAN	653	653	54.2 ± 18.5	49.35%	1156 ± 128
IXI	581	581	48.65 ± 16.47	53.87%	1137 ± 131
NACC	6956	4926	71.41 ± 11.25	53.34%	1034 ± 185
ADNI	6471	1817	74.68 ± 7.64	47.45%	1072 ± 116
AIBL	1167	664	74.56 ± 6.79	52.87%	1078 ± 110
OASIS1	436	416	51.35 ± 25.27	61.47%	1089 ± 153
OASIS2	373	150	77.01 ± 7.64	57.10%	1045 ± 124
OASIS3	2275	1044	70.69 ± 9.18	48.39%	1061 ± 113
OASIS4	181	180	71.09 ± 9.60	46.96%	1060 ± 116
MIRIAD	705	69	69.65 ± 6.88	55.51%	1039 ± 115
PPMI	2032	1306	63.29 ± 9.58	40.59%	1119 ± 157
OpenNeuro	163	163	6.67 ± 2.47	46.01%	1195 ± 144
EPISURG	268	268	NA	NA	1102 ± 133
UCSF-PDGM	1980	501	56.87 ± 15.02	40.32%	NA
BraTS	2502	1251	NA	NA	1103 ± 136
ATLAS	655	33	NA	NA	1445 ± 124

## Data Availability

Data from UK Biobank, ADNI, NACC, AIBL, OASIS 1–4, MIRIAD, PPMI, HCP, CamCAN, IXI, OpenNeuro, EPISURG, UCSF-PDGM, BraTS, and ATLAS are available to qualified researchers through application to the respective consortia (see Appendix A for links and access details). No new data were generated for this study.

## Code availability

The code used to train and evaluate the models described in this study is publicly available at https://github.com/MIQUEL-LOPEZ-ESCORIZA/ai4brainmri.
