# Supplementary material for: Comparative Study of Anatomical and Learned Features in AI Models for Structural Brain MRI
Source: Res Sq. 2026 Sep 9:rs.3.rs-10851563. Preprint. [Version 1] doi: 10.21203/rs.3.rs-10851563/v1 (PMC13622820; doi:10.21203/rs.3.rs-10851563/v1)
Supplement: Supplementary file 1 [file NIHPPrs10851563v1-supplement-1.pdf]

Supplementary information

Appendix A Dataset Information

A.1 Acknowledgement

This research has been conducted using the UK Biobank Resource under Application Number 166231. Research reported in this publication was supported by the National Institute On Aging of the National Institutes of Health under Award Number U01AG052564 and by funds provided by the McDonnell Center for Systems Neuroscience at Washington University in St. Louis. The HCP-Aging 2.0 Release data used in this report came from DOI: 10.15154/1520707

Data collection and sharing for this project was provided by the Cambridge Centre for Ageing and Neuroscience (CamCAN). CamCAN funding was provided by the UK Biotechnology and Biological Sciences Research Council (grant number BB/H008217/1), together with support from the UK Medical Research Council and University of Cambridge, UK. We acknowledge the use of data from the IXI dataset (https://brain-development.org/ixi-dataset/), which was made available through the Imaging in eXcellence Initiative (EPSRC GR/S21533/02).

The NACC database is funded by NIA/NIH Grant U24 AG072122. SCAN is a multi-institutional project that was funded as a U24 grant (AG067418) by the National

Institute on Aging in May 2020. Data collected by SCAN and shared by NACC are contributed by the NIA-funded ADRCs as follows:

Arizona Alzheimer's Center - P30 AG072980 (PI: Eric Reiman, MD); R01 AG069453 (PI: Eric Reiman (contact), MD); P30 AG019610 (PI: Eric Reiman, MD); and the State of Arizona which provided additional funding supporting our center; Boston University - P30 AG013846 (PI Neil Kowall MD); Cleveland ADRC - P30 AG062428 (James Leverenz, MD); Cleveland Clinic, Las Vegas – P20AG068053; Columbia - P50 AG008702 (PI Scott Small MD); Duke/UNC ADRC – P30 AG072958; Emory University - P30AG066511 (PI Levey Allan, MD, PhD); Indiana University - R01 AG19771 (PI Andrew Saykin, PsyD); P30 AG10133 (PI Andrew Saykin, PsyD); P30 AG072976 (PI Andrew Saykin, PsyD); R01 AG061788 (PI Shannon Risacher, PhD); R01 AG053993 (PI Yu-Chien Wu, MD, PhD); U01 AG057195 (PI Liana Apostolova, MD); U19 AG063911 (PI Bradley Boeve, MD); and the Indiana University Department of Radiology and Imaging Sciences; Johns Hopkins - P30 AG066507 (PI Marilyn Albert, Phd.); Mayo Clinic - P50 AG016574 (PI Ronald Petersen MD PhD); Mount Sinai - P30 AG066514 (PI Mary Sano, PhD); R01 AG054110 (PI Trey Hedden, PhD); R01 AG053509 (PI Trey Hedden, PhD); New York University - P30AG066512-01S2 (PI Thomas Wisniewski, MD); R01AG056031 (PI Ricardo Osorio, MD); R01AG056531 (PIs Ricardo Osorio, MD; Girardin Jean-Louis, PhD); Northwestern University - P30 AG013854 (PI Robert Vassar PhD); R01 AG045571 (PI Emily Rogalski, PhD); R56 AG045571, (PI Emily Rogalski, PhD); R01 AG067781, (PI Emily Rogalski, PhD); U19 AG073153, (PI Emily Rogalski, PhD); R01 DC008552, (M.-Marsel Mesulam, MD); R01 AG077444, (PIs M.-Marsel Mesulam, MD, Emily Rogalski, PhD); R01 NS075075 (PI Emily Rogalski, PhD); R01 AG056258 (PI Emily Rogalski, PhD); Oregon Health and Science University - P30 AG008017 (PI Jeffrey Kaye MD); R56 AG074321 (PI Jeffrey Kaye, MD); Rush University - P30 AG010161 (PI David Bennett MD); Stanford – P30AG066515; P50 AG047366 (PI Victor Henderson MD MS); University of Alabama, Birmingham – P20; University of California, Davis - P30 AG10129 (PI Charles DeCarli, MD); P30 AG072972 (PI Charles DeCarli, MD); University of California, Irvine - P50 AG016573 (PI Frank LaFerla PhD); University of California, San Diego - P30AG062429 (PI James Brewer, MD, PhD); University of California, San Francisco - P30 AG062422 (Rabinovici, Gil D., MD); University of Kansas - P30 AG035982 (Russell Swerdlow, MD); University of Kentucky - P30 AG028283-15S1 (PIs Linda Van Eldik, PhD and Brian Gold, PhD); University of Michigan ADRC - P30AG053760 (PI Henry Paulson, MD, PhD) P30AG072931 (PI Henry Paulson, MD, PhD) Cure Alzheimer's Fund 200775 - (PI Henry Paulson, MD, PhD) U19 NS120384 (PI Charles DeCarli, MD, University of Michigan Site PI Henry Paulson, MD, PhD) R01 AG068338 (MPI Bruno Giordani, PhD, Carol Persad, PhD, Yi Murphey, PhD) S10OD026738-01 (PI Douglas Noll, PhD) R01 AG058724 (PI Benjamin Hampstead, PhD) R35 AG072262 (PI Benjamin Hampstead, PhD) W81XWH2110743 (PI Benjamin Hampstead, PhD) R01 AG073235 (PI Nancy Chiaravalloti, University of Michigan Site PI Benjamin Hampstead, PhD) 1I01RX001534 (PI Benjamin Hampstead, PhD) IRX001381 (PI Benjamin Hampstead, PhD); University of New Mexico - P20 AG068077 (Gary Rosenberg, MD); University of Pennsylvania - State of PA project 2019NF4100087335 (PI David Wolk, MD);

Rooney Family Research Fund (PI David Wolk, MD); R01 AG055005 (PI David Wolk, MD); University of Pittsburgh - P50 AG005133 (PI Oscar Lopez MD); University of Southern California - P50 AG005142 (PI Helena Chui MD); University of Washington - P50 AG005136 (PI Thomas Grabowski MD); University of Wisconsin - P50 AG033514 (PI Sanjay Asthana MD FRCP); Vanderbilt University – P20 AG068082; Wake Forest - P30AG072947 (PI Suzanne Craft, PhD); Washington University, St. Louis - P01 AG03991 (PI John Morris MD); P01 AG026276 (PI John Morris MD); P20 MH071616 (PI Dan Marcus); P30 AG066444 (PI John Morris MD); P30 NS098577 (PI Dan Marcus); R01 AG021910 (PI Randy Buckner); R01 AG043434 (PI Catherine Roe); R01 EB009352 (PI Dan Marcus); UL1 TR000448 (PI Brad Evanoff); U24 RR021382 (PI Bruce Rosen); Avid Radiopharmaceuticals / Eli Lilly; Yale - P50 AG047270 (PI Stephen Strittmatter MD PhD); R01AG052560 (MPI: Christopher van Dyck, MD; Richard Carson, PhD); R01AG062276 (PI: Christopher van Dyck, MD); 1Florida - P30AG066506-03 (PI Glenn Smith, PhD); P50 AG047266 (PI Todd Golde MD PhD)

Data collection and sharing for this project was funded by the Alzheimer's Disease Neuroimaging Initiative (ADNI) (National Institutes of Health Grant U01 AG024904) and DOD ADNI (Department of Defense award number W81XWH-12-2-0012). ADNI is funded by the National Institute on Aging, the National Institute of Biomedical Imaging and Bioengineering, and through generous contributions from the following: AbbVie, Alzheimer's Association; Alzheimer's Drug Discovery Foundation; Araclon Biotech; BioClinica, Inc.; Biogen; Bristol-Myers Squibb Company; CereSpir, Inc.; Cogstate; Eisai Inc.; Elan Pharmaceuticals, Inc.; Eli Lilly and Company; EuroImmun; F. Hoffmann-La Roche Ltd and its affiliated company Genentech, Inc.; Fujirebio; GE Healthcare; IXICO Ltd.; Janssen Alzheimer Immunotherapy Research & Development, LLC.; Johnson & Johnson Pharmaceutical Research & Development LLC.; Lumosity; Lundbeck; Merck & Co., Inc.; Meso Scale Diagnostics, LLC.; NeuroRx Research; Neurotrack Technologies; Novartis Pharmaceuticals Corporation; Pfizer Inc.; Piramal Imaging; Servier; Takeda Pharmaceutical Company; and Transition Therapeutics. The Canadian Institutes of Health Research is providing funds to support ADNI clinical sites in Canada. Private sector contributions are facilitated by the Foundation for the National Institutes of Health (www.fnih.org). The grantee organization is the Northern California Institute for Research and Education, and the study is coordinated by the Alzheimer's Therapeutic Research Institute at the University of Southern California. ADNI data are disseminated by the Laboratory for Neuro Imaging at the University of Southern California.

Data were provided in part by OASIS. Specifically: OASIS-1: Cross-Sectional: Principal Investigators: D. Marcus, R. Buckner, J. Csernansky, J. Morris; P50 AG05681, P01 AG03991, P01 AG026276, R01 AG021910, P20 MH071616, U24 RR021382.

OASIS-2: Longitudinal: Principal Investigators: D. Marcus, R. Buckner, J. Csernansky, J. Morris; P50 AG05681, P01 AG03991, P01 AG026276, R01 AG021910, P20 MH071616, U24 RR021382.

OASIS-3: Longitudinal Multimodal Neuroimaging:

Principal Investigators: T. Benzinger, D. Marcus, J. Morris; NIH P30 AG066444, P50 AG00561, P30 NS09857781, P01 AG026276, P01 AG003991, R01 AG043434, UL1

TR000448, R01 EB009352. AV-45 doses were provided by Avid Radiopharmaceuticals, a wholly owned subsidiary of Eli Lilly.

OASIS-3_AV1451:

Principal Investigators: T. Benzinger, J. Morris; NIH P30 AG066444, AW00006993. AV-1451 doses were provided by Avid Radiopharmaceuticals, a wholly owned subsidiary of Eli Lilly.

OASIS-4: Clinical Cohort: Principal Investigators: T. Benzinger, L. Koenig, P. LaMontagne.

Data used in the preparation of this article were obtained from the MIRIAD database. The MIRIAD investigators did not participate in analysis or writing of this report. The MIRIAD dataset is made available through the support of the UK Alzheimer's Society (Grant RF116). The original data collection was funded through an unrestricted educational grant from GlaxoSmithKline (Grant 6GKC).

Data used in the preparation of this article was obtained on 2023-11-30 from the Parkinson's Progression Markers Initiative (PPMI) database, RRID:SCR_006431. For up-to-date information on the study, visit www.ppmi-info.org.

PPMI – a public-private partnership – is funded by the Michael J. Fox Foundation for Parkinson's Research and funding partners, including 4D Pharma, Abbvie, AcureX, Allergan, Amathus Therapeutics, Aligning Science Across Parkinson's, AskBio, Avid Radiopharmaceuticals, BIAL, BioArctic, Biogen, Biohaven, BioLegend, BlueRock Therapeutics, Bristol-Myers Squibb, Calico Labs, Capsida Biotherapeutics, Celgene, Cerevel Therapeutics, Coave Therapeutics, DaCapo Brainscience, Denali, Edmond J. Safra Foundation, Eli Lilly, Gain Therapeutics, GE HealthCare, Genentech, GSK, Golub Capital, Handl Therapeutics, Insitro, Jazz Pharmaceuticals, Johnson & Johnson Innovative Medicine, Lundbeck, Merck, Meso Scale Discovery, Mission Therapeutics, Neurocrine Biosciences, Neuron23, Neuropore, Pfizer, Piramal, Prevail Therapeutics, Roche, Sanofi, Servier, Sun Pharma Advanced Research Company, Takeda, Teva, UCB, Vanqua Bio, Verily, Voyager

We also highly appreciate the BraTS and ATLAS organizers for holding the great challenge, and creating the publicly available dataset.

We also highly appreciate BioRender team (https://BioRender.com) for making vivid icons available.

A.2 Pretraining Dataset

UK Biobank (UKBB)[18]: The UK Biobank study is a large-scale population-based study that includes a diverse collection of health, demographic, and lifestyle information to explore aging, disease, and cognitive functioning. It provides rich neuroimaging data for studying brain structure, function, and their relationship with various diseases. Our study focused on participants from the imaging-genomics population who had at least one brain MRI scan. UKBB acquired T1-weighted brain MRI data using 3T Siemens Skyra MRI scanner and the Magnetization Prepared Rapid Gradient Echo (MPRAGE) sequence. Some participants (7.79% in our data freeze) receive a second scan within 2 years after their initial scan. Our collection is based on data available in March, 2024.

Human Connectome Project (HCP)[47, 48]: The Human Connectome Project Lifespan Project focused on gathering data from healthy adults to build a high-quality dataset for comparability with other populations. The HCP-Aging (age 36-60 years) and HCP-Development (age 5-21 years) investigate healthy aging at different development stages. All imaging follow the same Siemens Prisma protocol. Current data release include cross-sectional visit 1 structural brain imaging data.

Cambridge Centre for Ageing and Neuroscience (CamCAN)[49]: Cambridge Centre for Ageing and Neuroscience investigates how healthy people retain cognitive abilities as they age. The project uses data from interviews, brain scans, and cognitive experiments. T1-weighted MRI scans are collected using a standardized protocol to ensure high-quality and consistent imaging across the participant cohort.

Information eXtraction from Images(IXI)[50]: Information eXtraction from Images project collected neuroimaging data from normal, healthy subjects with varying scanners at three different hospitals in London. All participants in the IXI study are healthy individuals, offering valuable data for understanding normal aging and brain structure changes, without the confounding effects of neurological conditions.

A.3 Disease Specific Datasets

Multiple neuroimaging datasets aim to identify imaging biomarkers for understanding the trajectory of cognitive decline. They differ in terms of study design, patient selection, patient tracking and image data collection protocol. The diversity of these datasets helps confirm the model's generalizability and reliability for use in diverse healthcare environments, ultimately improving the model's ability to support early and accurate diagnosis of Alzheimer's disease.

National Alzheimer's Coordinating Center (NACC)[20]: NACC study is a multi-source collection which provides longitudinal data to support research into Alzheimer's disease, including clinical, genetic, cognitive assessment and imaging data. The project actively follows participants. We include all visits for all participants as of the June 2023 data freeze. The NACC dataset includes MRI scans collected with mixed protocols, including variations in scanner models, acquisition parameters, and protocols. This diversity enables the evaluation of model robustness by testing how well models generalize across different imaging conditions and real-world data variability.

Alzheimer's Disease Neuroimaging Initiative (ADNI)[19]: Data used in the preparation of this article were obtained from the Alzheimer's Disease Neuroimaging Initiative (ADNI) database (adni.loni.usc.edu). The ADNI was launched in 2003 as a public-private partnership, led by Principal Investigator Michael W. Weiner, MD. The primary goal of ADNI has been to test whether serial magnetic resonance imaging (MRI), positron emission tomography (PET), other biological markers, and clinical and neuropsychological assessment can be combined to measure the progression of mild cognitive impairment (MCI) and early Alzheimer's disease (AD). We include all available T1 structural MRI images for all participants as of the October 2023 data freeze.

OASIS (Open Access Series of Imaging Studies)[23–25]: The Open Access Series of Imaging Studies (OASIS) dataset primarily targets abnormal aging and Alzheimer's Disease. It includes a variety of studies with different focuses and includes

different participant cohorts. The previously released OASIS1 (cross-sectional) and OASIS2 (longitudinal) datasets have been used for hypothesis-driven analyses, the development of neuroanatomical atlases, and the creation of segmentation algorithms. OASIS-3 is a longitudinal dataset that includes multimodal neuroimaging, clinical, cognitive, and biomarker data focused on normal aging and Alzheimer's Disease. OASIS-4 consists of MRI, clinical, cognitive, and biomarker data from individuals with memory complaints. Each part is analyzed separately in our study. We include 436 / 373 / 2275 / 181 MRI images (416 / 150 / 1044 / 180 unique subjects) from OASIS1, OASIS2, OASIS3, OASIS4 study.

Australian Imaging, Biomarkers & Lifestyle (AIBL)[21]: Data used in the preparation of this article was obtained from the Australian Imaging Biomarkers and Lifestyle flagship study of ageing (AIBL). See www.aibl.csiro.au for further details. We include available T1 MRI images from the AIBL study, which are made available via LONI (Laboratory of Neuroimaging, USC) infrastructure.

Mild Cognitive Impairment and Alzheimer's Disease Neuroimaging Database (MIRIAD)[22]: We include 705 T1 MRI images (of 69 unique subjects) from the MIRIAD study, which focuses on the transition from mild cognitive impairment (MCI) to Alzheimer's disease, including neuroimaging and cognitive data. All scans were conducted on the same 1.5 T Signa MRI scanner (GE Medical systems, Milwaukee, WI) and acquired by the same radiographer.

Parkinson's Progression Markers Initiative (PPMI)[26]: Parkinson's Progression Markers Initiative dataset investigates biomarkers of Parkinson's disease through imaging, clinical, and biological data to better understand disease progression. Healthy controls only received imaging as baseline visit, prodromal and Parkinson's Disease patients might have multiple images. We include data from PPMI participants available in November 2023.

OpenNeuro[28]: An MRI dataset of 82 people with epilepsy due to focal cortical dysplasia (FCD) type II and 81 healthy controls was made available in OpenNeuro platform (ds004199). Focal cortical dysplasias (FCDs) are the most common cause of treatment-resistant epilepsy affecting the pediatric population. Most individuals with FCD have seizure onset within the first five years of life and the majority will develop epilepsy by the age of sixteen. We use all T1 MRI scans to develop a model that automatically detects FCD thus identifies epilepsy.

EPISURG[29]: EPISURG is a clinical dataset of T1-weighted magnetic resonance images (MRI) from 430 epileptic patients who underwent resective brain surgery. All preoperative scans available (n=268) were included in this study as an external validation of model performance.

University of California San Francisco Preoperative Diffuse Glioma (UCSF-PDGM)[27]. The publicly available MRI dataset, consisting of 501 patients with grade 2–4 diffuse gliomas, includes standardized 3-T three-dimensional preoperative MRI protocol neuroimaging data, tumor genetic data, and treatment and survival data. We focus on T1 without and with contrast enhancing(CE) agent, as well as the tumor genetic data for our analysis.

Brain Tumor Segmentation (BraTS) Continuous Challenge[30–32]: The challenge seeks to identify state-of-the-art segmentation algorithms for brain diffuse glioma patients and their sub-regions. We include T1 MRI scans from BraTS 2023 Adult Glioma challenge training cohort. Each subject received two T1 scans, one with contrast enhancing(CE) agent, one without. All the imaging datasets have been annotated manually by experienced neuro-radiologists. Annotations comprise the enhancing tumor (ET — label 3), the peritumoral edematous/invaded tissue (ED — label 2), and the necrotic tumor core (NCR — label 1). Enhancing tumour refers to regions with visible enhancement on a T1CE sequence after gadolinium administration. Non-enhancing tumour/necrotic tumour core refers to the part of the tumour that does not enhance after gadolinium, typically deep to the enhancement, while oedema/invaded tissue refers to the peritumoral oedematous and/or infiltrated brain parenchyma, typified by hyperintensity on T2 and FLAIR sequences.

Anatomical Tracings of Lesions After Stroke(ATLAS R2.0)[33]: The goal is to discover automated methods of stroke lesion segmentation in MR images. We include 655 T1 MRI images (of 33 unique subjects) and their manual segmentation masks from the public training collection of the 2022 MICCAI ATLAS challenge.

This BrainATLAS collection is one of the most diverse and comprehensive public brain MRI datasets, encompassing a wide range of demographics, conditions, and imaging protocols. Its breadth and diversity offer significant potential for advancing AI-driven brain research and enhancing clinical decision-making across various neurological conditions. We provide detailed study population characteristics for each individual dataset in Appendix Table A1. A complete list of data usage acknowledgement for can also be found in Appendix Section A.1.

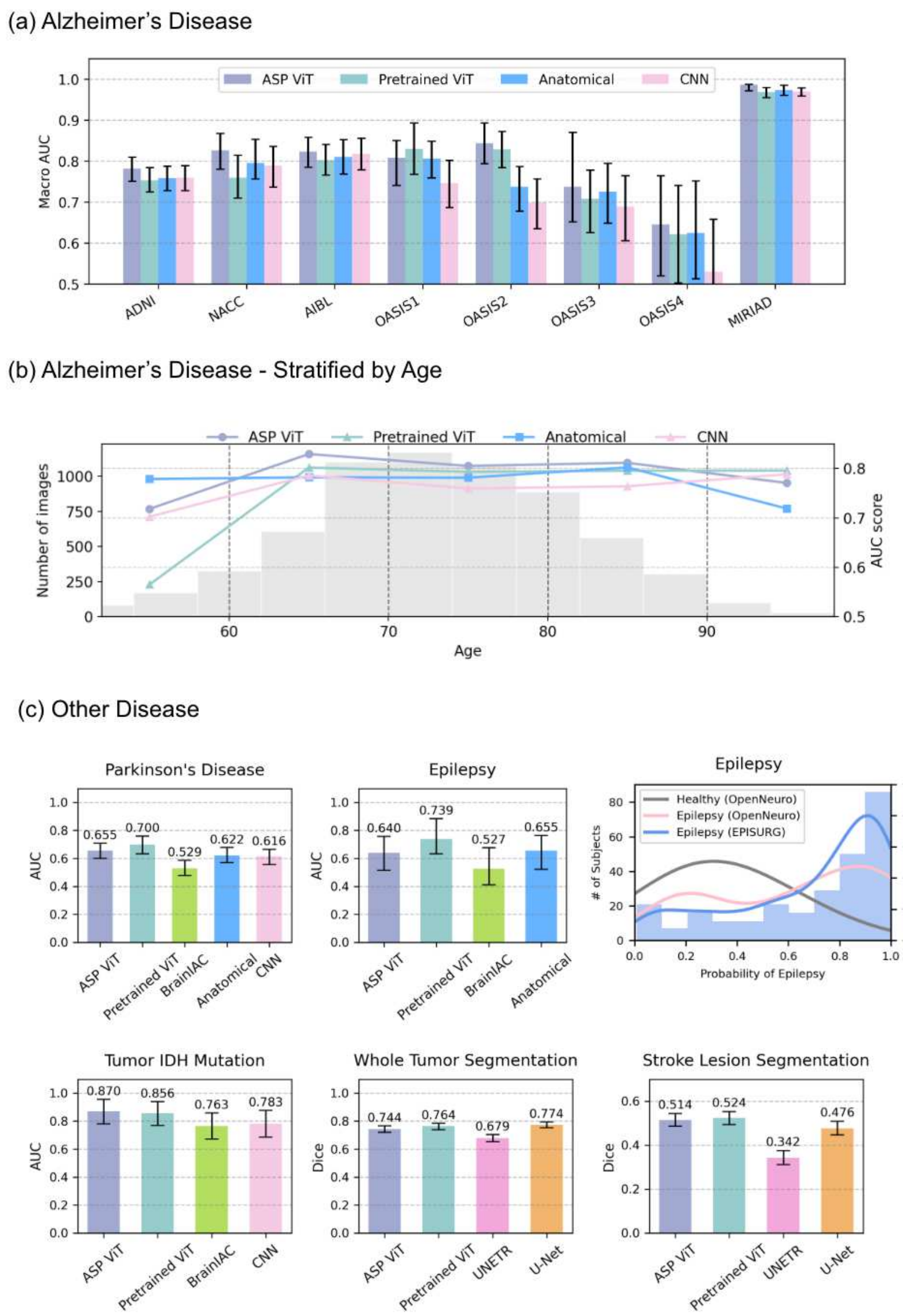

Fig. 2 Performance comparison across diagnostic and segmentation tasks. Alzheimer's disease diagnosis was evaluated across an internal ADNI test cohort and seven external cohorts (NACC, AIBL, MIRIAD, and OASIS 1-4), where ASP ViT achieved the highest macro-AUC in most cohorts, though statistical significance over the CNN and anatomical logistic regression was only reached for OASIS 2. In the diagnostics of Parkinson's disease (PPMI) and Epilepsy (OpenNeuro), the Pretrained ViT reached the highest AUC, showing a statistically significant improvement only over BrainIAC. Estimated probabilities for Epilepsy from ASP ViT are also shown for EPISURG, an additional evaluation set with only epilepsy cases. For the identification of brain tumor IDH mutations (UCSF-PDGM) ASP ViT yielded the highest AUC, though these differences were not statistically significant. CNN could not be applied to the epilepsy task due to limited training data. In segmentation tasks, a U-Net achieved the highest Dice score for adult brain gliomas (BraTS 2023), while the Pretrained ViT performed best for stroke lesion segmentation (ATLAS R2.0); in both cases, the differences were only statistically significant relative to UNETR. Overall, no significant performance differences were observed between the pretrained ViTs, CNNs, and anatomical logistic regression models across the evaluated tasks.

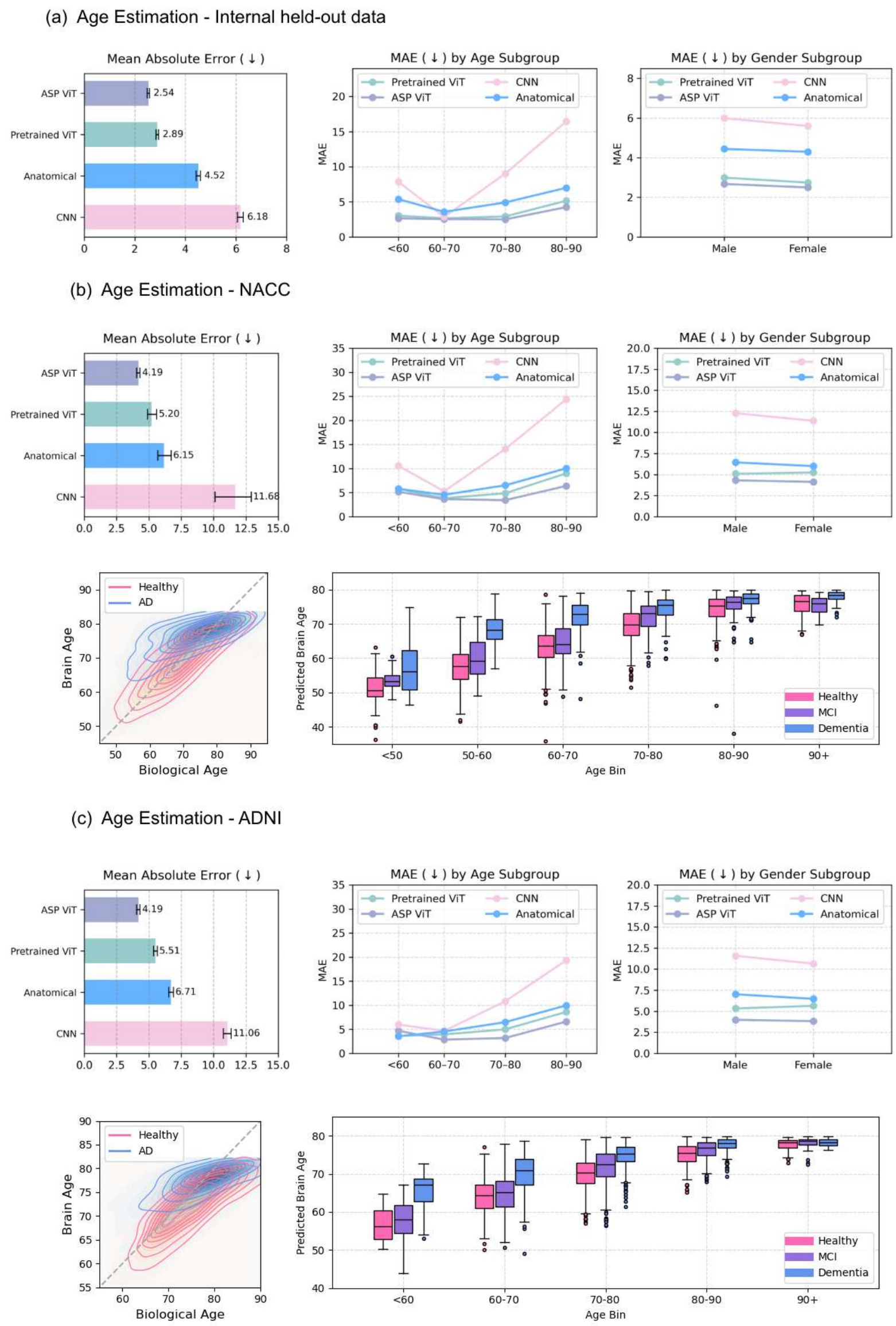

Fig. 3 Age estimation performance across internal and external cohorts. The ASP ViT model demonstrates superior performance, achieving a mean absolute error (MAE) of 2.54 years in the internal test cohort (a) and 4.19 years in both the NACC (b) and ADNI (c) external test cohorts. While the Pretrained ViT maintains competitive performance on the internal cohort with an MAE only 0.35 years greater than the ASP ViT, this performance gap widens to over 1 year in the external cohorts. Logistic regression underperforms relative to the ViT-based approaches by a margin of 1 to 2 years, though it consistently outperforms the CNN. These performance comparisons remain stable when the data is stratified by sex and age. Age-based stratification reveals more pronounced errors for individuals over 80, a demographic less represented in the training and pretraining datasets. When the best-performing model (ASP ViT) is applied to clinical populations, age estimates for dementia patients from the NACC (b) and ADNI (c) datasets are consistently higher than their biological age across most age intervals.

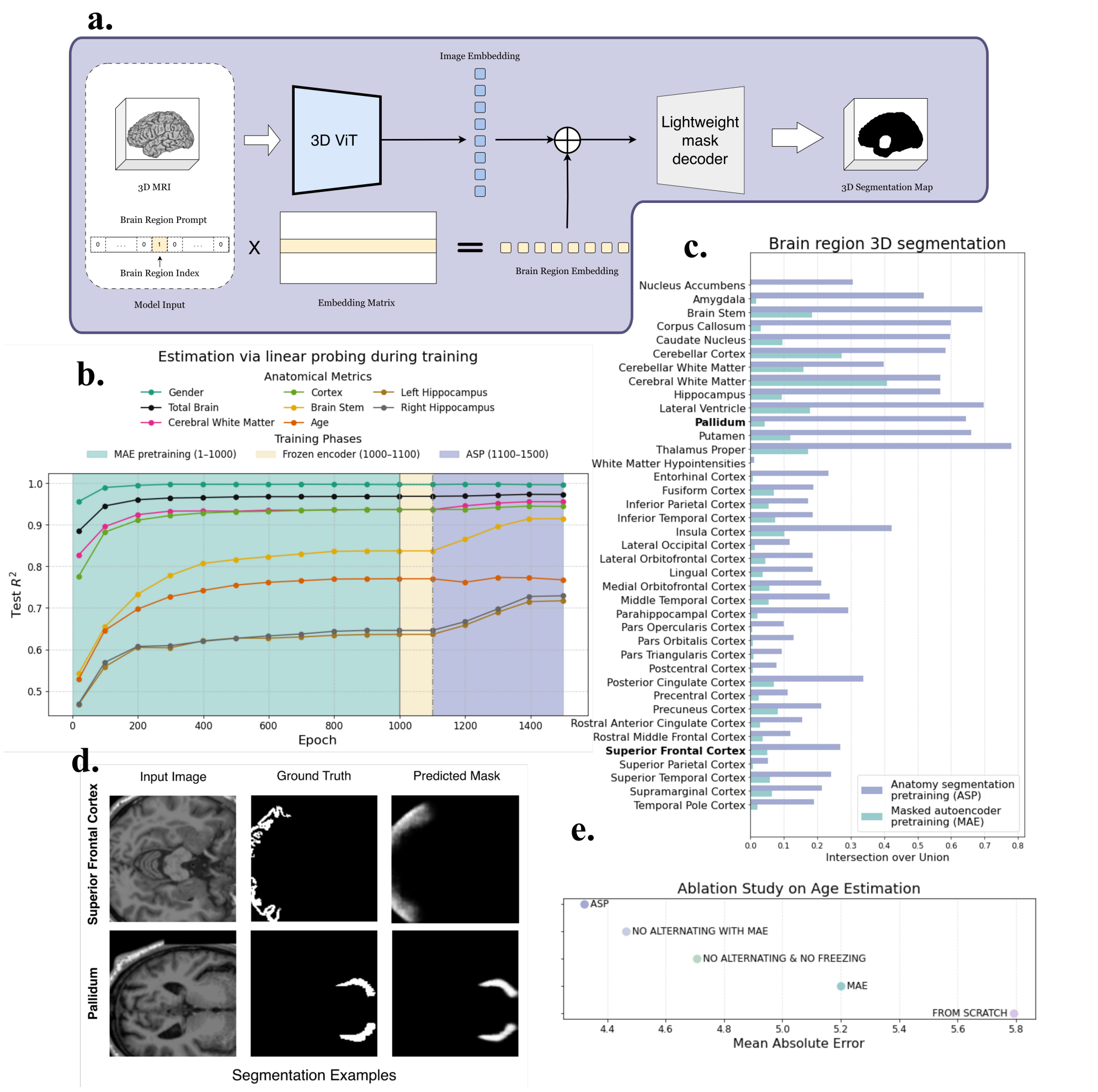

Fig. 4 **a. Prompt-based segmentation architecture**. The input consists of an image and a brain region index, which is used to retrieve the corresponding brain region embedding. This embedding is added to the image representation, and the combined features are passed through a lightweight decoder to predict the segmentation mask for the specified region. **b. Linear-probe accuracy/R^2** on six target variables—two global attributes (age, gender) and four region-specific volumetric measures—tracked over pre-training epochs. The differences in overall accuracy across regions arise because some regions are inherently easier to predict than others. For example, cortex volume tends to be more accurately segmented due to its larger size and higher contrast, compared to smaller and less distinct regions like the hippocampus. For demographic statistics the same happens. Gender is easier to infer than age since it is a binary attribute, while age requires finer-grained regression. **c–d. Validation IoU by Brain Region.** Regional performance varies based on anatomical complexity; for instance, the superior frontal cortex's small, irregular geometry results in lower IoU, whereas the smoother, more consistent morphology of the pallidum allows for higher segmentation accuracy. **e. ASP training ablation.** Age-estimation performance for different ASP training variants on the external NACC cohort. The full ASP model achieves the lowest error, while removing alternating MAE updates, removing encoder freezing, using MAE alone, or training from scratch reduces performance. Detailed results are provided in Appendix Section C.3.

A.4 Study Population in Each Dataset

We present summary statistics regarding participant demographic and imaging acquisition protocols in Table A1. The BrainATLAS represents one of the most diverse and comprehensive collections of publicly available brain MRI data, drawn from 18 studies. Its extensive breadth and variability make it a valuable resource for advancing research in brain imaging and improving the generalizability of AI models in clinical applications.

**Dataset**	# images	# subjects	Age in yrs (mean ± std)	Gender (female %)	Brain volume in mL (mean ± std)
UKBB	67294	62665	64.93 ± 7.69	52.81%	1156 ± 114
HCP	1350	1350	38.66 ± 25.76	54.97%	1179 ± 137
CamCAN	653	653	54.2 ± 18.5	49.35%	1156 ± 128
IXI	581	581	48.65 ± 16.47	53.87%	1137 ± 131
NACC	6956	4926	71.41 ± 11.25	53.34%	1034 ± 185
ADNI	6471	1817	74.68 ± 7.64	47.45%	1072 ± 116
AIBL	1167	664	74.56 ± 6.79	52.87%	1078 ± 110
OASIS1	436	416	51.35 ± 25.27	61.47%	1089 ± 153
OASIS2	373	150	77.01 ± 7.64	57.10%	1045 ± 124
OASIS3	2275	1044	70.69 ± 9.18	48.39%	1061 ± 113
OASIS4	181	180	71.09 ± 9.60	46.96%	1060 ± 116
MIRIAD	705	69	69.65 ± 6.88	55.51%	1039 ± 115
PPMI	2032	1306	63.29 ± 9.58	40.59%	1119 ± 157
OpenNeuro	163	163	6.67 ± 2.47	46.01%	1195 ± 144
EPISURG	268	268	NA	NA	1102 ± 133
UCSF-PDGM	1980	501	56.87 ± 15.02	40.32%	NA
BraTS	2502	1251	NA	NA	1103 ± 136
ATLAS	655	33	NA	NA	1445 ± 124

Footnotes:

- Excluding missing values and outliers.
- Brain volume: BrainSegVol generated by FreeSurfer.
- NA: Not available for the analyzed cohort..

Table A1 Dataset distribution in BrainATLAS. We include a total of 18 distinct neuroimaging dataset from diverse populations.

A.5 Imaging Data Harmonization

There are a total of 31 steps in the workflow. We conduct a retrospective analysis for using 1500 successful image processing logs to analyze the computational cost for running "recon-all" command in FreeSurfer. We provide a summary of time consumption for different groups of operations in Table A2. On average, the workflow takes 6.11 hours to finish for each 3D image.

1. Motion Correction and Conform
2. NU (Non-Uniform intensity normalization)
3. Talairach transform computation
4. Intensity Normalization 1
5. Skull Strip
6. EM Register (linear volumetric registration)
7. CA Intensity Normalization
8. CA Non-linear Volumetric Registration
9. Remove Neck
10. LTA with Skull
11. CA Label (Volumetric Labeling, ie Aseg) and Statistics
12. Intensity Normalization 2 (start here for control points)
13. White matter segmentation
14. Edit WM With ASeg
15. Fill (start here for wm edits)
16. Tessellation (begins per-hemisphere operations)
17. Smooth1
18. Inflate1
19. QSphere
20. Automatic Topology Fixer
21. Final Surfs (start here for brain edits for pial surf)
22. Smooth2
23. Inflate2
24. Spherical Mapping
25. Spherical Registration
26. Spherical Registration, Contralateral hemisphere
27. Map average curvature to subject
28. Cortical Parcellation - Desikan_Killiany and Christophe (Labeling)
29. Cortical Parcellation Statistics
30. Cortical Ribbon Mask
31. Cortical Parcellation mapping to Aseg

	**Workflow**	**Explanation**	**Time (mean ± std, hours)**
**-autorecon1**	steps 1-5	Initial processing of the MRI data	0.36 ± 0.06
**-autorecon2**	steps 6-23	Reconstruction of the brain's cortical surface and refinement of subcortical structures	3.78 ± 0.76
**-autorecon3**	steps 24-31	Refinement of segmentation and cortical surfaces	1.97 ± 0.39

Table A2 FreeSurfer main workflow explanation and processing time

A.6 Brain Anatomical Features

We extract 178 anatomical brain features using FreeSurfer output statistics regarding brain segmentation and surface reconstruction. We summarize the statistical features in Table A3. These features are categorized into *Volume*, *Surface Area*, and *Thickness*, and collectively capture key structural information of the brain.

**Category**	**Feature Name**
Volume	Right-VentralDC_Volume_mm3
Volume	CC_Posterior_Volume_mm3
Volume	3rd-Ventricle_Volume_mm3
Volume	inferiortemporal_GrayVol
Volume	lateraloccipital_GrayVol
Volume	lateralorbitofrontal_GrayVol
Volume	CC_Mid_Posterior_Volume_mm3
Volume	paracentral_GrayVol
Volume	VentricleChoroidVol
Volume	rostralanteriorcingulate_GrayVol
Volume	middletemporal_GrayVol
Volume	Left-Amygdala_Volume_mm3
Volume	TotalGrayVol
Volume	CortexVol
Volume	fusiform_GrayVol
Volume	precentral_GrayVol
Volume	transversetemporal_GrayVol
Volume	BrainSegVolNotVent
Volume	supramarginal_GrayVol
Volume	Left-Putamen_Volume_mm3
Volume	parsopercularis_GrayVol
Volume	Left-vessel_Volume_mm3
Volume	Brain-Stem_Volume_mm3
Volume	Right-Amygdala_Volume_mm3
Volume	entorhinal_GrayVol
Volume	5th-Ventricle_Volume_mm3
Volume	BrainSegVol
Volume	insula_GrayVol
Volume	Right-Thalamus_Volume_mm3
Volume	Right-vessel_Volume_mm3
Volume	Right-Inf-Lat-Vent_Volume_mm3
Volume	Right-choroid-plexus_Volume_mm3
Volume	Left-Hippocampus_Volume_mm3
Volume	CC_Mid_Anterior_Volume_mm3
Volume	caudalanteriorcingulate_GrayVol
Volume	Right-Caudate_Volume_mm3
Volume	lingual_GrayVol
Volume	SupraTentorialVolNotVent
Volume	Optic-Chiasm_Volume_mm3
Volume	Left-Accumbens-area_Volume_mm3

(continued on next page)

**Category**	**Feature Name**
Volume	posteriorcingulate_GrayVol
Volume	bankssts_GrayVol
Volume	rhCortexVol
Volume	parahippocampal_GrayVol
Volume	Right-Cerebellum-Cortex_Volume_mm3
Volume	Left-Thalamus_Volume_mm3
Volume	Right-Accumbens-area_Volume_mm3
Volume	Left-Cerebellum-Cortex_Volume_mm3
Volume	temporalpole_GrayVol
Volume	parstriangularis_GrayVol
Volume	Right-Hippocampus_Volume_mm3
Volume	superiorparietal_GrayVol
Volume	4th-Ventricle_Volume_mm3
Volume	pericalcarine_GrayVol
Volume	cuneus_GrayVol
Volume	Left-choroid-plexus_Volume_mm3
Volume	superiorfrontal_GrayVol
Volume	Right-Lateral-Ventricle_Volume_mm3
Volume	WM-hypointensities_Volume_mm3
Volume	rostralmiddlefrontal_GrayVol
Volume	Left-Caudate_Volume_mm3
Volume	CerebralWhiteMatterVol
Volume	Left-Pallidum_Volume_mm3
Volume	Left-Inf-Lat-Vent_Volume_mm3
Volume	lhCerebralWhiteMatterVol
Volume	CC_Anterior_Volume_mm3
Volume	CC_Central_Volume_mm3
Volume	inferiorparietal_GrayVol
Volume	caudalmiddlefrontal_GrayVol
Volume	CSF_Volume_mm3
Volume	Left-Lateral-Ventricle_Volume_mm3
Volume	isthmuscingulate_GrayVol
Volume	parsorbitalis_GrayVol
Volume	postcentral_GrayVol
Volume	Right-Cerebellum-White-Matter_Volume_mm3
Volume	Left-Cerebellum-White-Matter_Volume_mm3
Volume	frontalpole_GrayVol
Volume	superiortemporal_GrayVol
Volume	Right-Putamen_Volume_mm3
Volume	precuneus_GrayVol
Volume	Left-VentralDC_Volume_mm3
Volume	Right-Pallidum_Volume_mm3
Volume	medialorbitofrontal_GrayVol
Volume	Left-WM-hypointensities_Volume_mm3
Volume	Right-WM-hypointensities_Volume_mm3
Volume	non-WM-hypointensities_Volume_mm3
Volume	Left-non-WM-hypointensities_Volume_mm3

(continued on next page)

**Category**	**Feature Name**
Volume	Right-non-WM-hypointensities_Volume_mm3
Volume	BrainSegVol-to-eTIV
Surface Area	transversetemporal_SurfArea
Surface Area	temporalpole_SurfArea
Surface Area	supramarginal_SurfArea
Surface Area	rostralmiddlefrontal_SurfArea
Surface Area	posteriorcingulate_SurfArea
Surface Area	parsorbitalis_SurfArea
Surface Area	parsopercularis_SurfArea
Surface Area	middletemporal_SurfArea
Surface Area	lingual_SurfArea
Surface Area	lateralorbitofrontal_SurfArea
Surface Area	caudalmiddlefrontal_SurfArea
Surface Area	caudalanteriorcingulate_SurfArea
Surface Area	bankssts_SurfArea
Surface Area	isthmuscingulate_SurfArea
Surface Area	insula_SurfArea
Surface Area	parahippocampal_SurfArea
Surface Area	parstriangularis_SurfArea
Surface Area	entorhinal_SurfArea
Surface Area	pericalcarine_SurfArea
Surface Area	paracentral_SurfArea
Surface Area	precentral_SurfArea
Surface Area	inferiortemporal_SurfArea
Surface Area	fusiform_SurfArea
Surface Area	cuneus_SurfArea
Surface Area	precuneus_SurfArea
Surface Area	lateraloccipital_SurfArea
Surface Area	supramarginal_SurfArea
Surface Area	superiorfrontal_SurfArea
Surface Area	inferiorparietal_SurfArea
Surface Area	superiorparietal_SurfArea
Surface Area	superiortemporal_SurfArea
Surface Area	rostralanteriorcingulate_SurfArea
Surface Area	superiororbitofrontal_SurfArea
Surface Area	postcentral_SurfArea
Surface Area	medialorbitofrontal_SurfArea
Surface Area	frontalpole_SurfArea
Surface Area	rhCortexArea
Thickness	insula_ThickAvg
Thickness	transversetemporal_ThickAvg
Thickness	temporalpole_ThickAvg
Thickness	frontalpole_ThickAvg
Thickness	superiortemporal_ThickAvg
Thickness	superiorparietal_ThickAvg
Thickness	superiorfrontal_ThickAvg

(continued on next page)

**Category**	**Feature Name**
Thickness	rostralmiddlefrontal_ThickAvg
Thickness	rostralanteriorcingulate_ThickAvg
Thickness	precuneus_ThickAvg
Thickness	precentral_ThickAvg
Thickness	posteriorcingulate_ThickAvg
Thickness	postcentral_ThickAvg
Thickness	pericalcarine_ThickAvg
Thickness	parstriangularis_ThickAvg
Thickness	parsorbitalis_ThickAvg
Thickness	parsopercularis_ThickAvg
Thickness	paracentral_ThickAvg
Thickness	parahippocampal_ThickAvg
Thickness	middletemporal_ThickAvg
Thickness	medialorbitofrontal_ThickAvg
Thickness	lingual_ThickAvg
Thickness	lateralorbitofrontal_ThickAvg
Thickness	lateraloccipital_ThickAvg
Thickness	isthmuscingulate_ThickAvg
Thickness	inferiortemporal_ThickAvg
Thickness	inferiorparietal_ThickAvg
Thickness	fusiform_ThickAvg
Thickness	entorhinal_ThickAvg
Thickness	cuneus_ThickAvg
Thickness	caudalmiddlefrontal_ThickAvg
Thickness	bankssts_ThickAvg
Thickness	caudalanteriorcingulate_ThickAvg
Thickness	rhCortexThickness
Thickness	CortexThickness
Thickness	lhCortexThickness
Thickness	supramarginal_CortexThickness
Thickness	parahippocampal_CortexThickness
Thickness	lateraloccipital_CortexThickness
Thickness	superiorparietal_CortexThickness
Thickness	precentral_CortexThickness
Thickness	medialorbitofrontal_CortexThickness
Thickness	isthmuscingulate_CortexThickness
Thickness	lingual_CortexThickness
Thickness	rostralmiddlefrontal_CortexThickness
Thickness	entorhinal_CortexThickness
Thickness	cuneus_CortexThickness
Thickness	temporalpole_CortexThickness
Thickness	postcentral_CortexThickness
Thickness	MeanThickness
Other	SurfaceHoles

Other	NumVert

Table A3: Brain anatomical features categorized by Volume, Surface Area, and Cortex Thickness.

A.7 Anatomical Brain Segmentation Workflow

In this section, we provide more detail into the anatomical brain segmentation workflow used to generate region-level labels from raw MRI volumes. All MRIs included in our pretraining dataset were processed using the FreeSurfer software suite, a well-established tool for structural neuroimaging analysis. FreeSurfer automatically segments the brain into detailed anatomical regions using both volumetric and surface-based techniques.

This segmentation provides a rich label space with 112 distinct anatomical regions, which we used as the basis for supervised learning. However, to make model training computationally feasible and clinically meaningful, we applied a post-processing pipeline described in the Methods section to reduce and simplify the label space. The goal was to balance granularity with practicality, preserving essential neuroanatomical information relevant to disease.

Appendix Table A4 presents the original anatomical regions segmented by FreeSurfer before any preprocessing. Each row corresponds to a distinct label from the raw segmentation. The Region column indicates the numeric label assigned by FreeSurfer, Num MRIs shows how many scans included that region, Percentage Brain Pixels represents the average proportion of brain volume it occupies, and Label provides the anatomical name.

Appendix Table A5 shows the final set of 39 grouped regions used for supervised training. These were obtained by merging symmetrical, related, or minor structures based on clinical relevance and volume thresholds. The Normalized Label column contains the name of each new grouped region, Percentage Brain Pixels indicates the average volume it occupies relative to the whole brain, and Region lists the original FreeSurfer label IDs that were combined.

**Region**	**Number or MRIs**	**Percentage Brain Pixels**	**Label**
2	66709	19.67	Left-Cerebral-White-Matter
4	66709	1.22	Left-Lateral-Ventricle
5	66706	0.04	Left-Inf-Lat-Vent
7	66709	1.29	Left-Cerebellum-White-Matter
8	66709	4.64	Left-Cerebellum-Cortex
10	66709	0.62	Left-Thalamus-Proper
11	66709	0.29	Left-Caudate
12	66709	0.39	Left-Putamen

(continued on next page)

Region	Number of MRIs	Percentage Brain Pixels	Label
13	66709	0.17	Left-Pallidum
14	66709	0.12	3rd-Ventricle
15	66709	0.15	4th-Ventricle
16	66709	1.88	Brain-Stem
17	66709	0.35	Left-Hippocampus
18	66709	0.13	Left-Amygdala
24	66709	0.10	CSF
26	66709	0.04	Left-Accumbens-area
28	66709	0.35	Left-VentralDC
30	64102	0.00	Left-vessel
31	66709	0.07	Left-choroid-plexus
41	66709	19.75	Right-Cerebral-White-Matter
43	66709	1.11	Right-Lateral-Ventricle
44	66708	0.04	Right-Inf-Lat-Vent
46	66709	1.23	Right-Cerebellum-White-Matter
47	66709	4.79	Right-Cerebellum-Cortex
49	66709	0.62	Right-Thalamus-Proper
50	66709	0.30	Right-Caudate
51	66709	0.40	Right-Putamen
52	66709	0.17	Right-Pallidum
53	66709	0.37	Right-Hippocampus
54	66709	0.15	Right-Amygdala
58	66709	0.05	Right-Accumbens-area
60	66709	0.35	Right-VentralDC
62	63318	0.00	Right-vessel
63	66709	0.07	Right-choroid-plexus
77	66709	0.21	WM-hypointensities
85	66707	0.02	Optic-Chiasm
251	66709	0.10	CC_Posterior
252	66709	0.05	CC_Mid_Posterior
253	66709	0.05	CC_Central
254	66709	0.05	CC_Mid_Anterior
255	66709	0.08	CC_Anterior
1001	66709	0.18	ctx-lh-bankssts
1002	66708	0.14	ctx-lh-caudalanteriorcingulate
1003	66709	0.51	ctx-lh-caudalmiddlefrontal
1005	66709	0.26	ctx-lh-cuneus
1006	66706	0.17	ctx-lh-entorhinal
1007	66709	0.79	ctx-lh-fusiform
1008	66709	0.94	ctx-lh-inferiorparietal
1009	66709	0.91	ctx-lh-inferiortemporal
1010	66709	0.22	ctx-lh-isthmuscingulate
1011	66709	1.02	ctx-lh-lateraloccipital
1012	66709	0.62	ctx-lh-lateralorbitofrontal
1013	66709	0.54	ctx-lh-lingual
1014	66708	0.44	ctx-lh-medialorbitofrontal
1015	66709	0.88	ctx-lh-middletemporal

(continued on next page)

Region	Number of MRIs	Percentage Brain Pixels	Label
1016	66705	0.18	ctx-lh-parahippocampal
1017	66708	0.29	ctx-lh-paracentral
1018	66709	0.37	ctx-lh-parsopercularis
1019	66709	0.19	ctx-lh-parsorbitalis
1020	66709	0.28	ctx-lh-parstriangularis
1021	66709	0.16	ctx-lh-pericalcarine
1022	66709	0.78	ctx-lh-postcentral
1023	66709	0.25	ctx-lh-posteriorcingulate
1024	66709	1.10	ctx-lh-precentral
1025	66709	0.78	ctx-lh-precuneus
1026	66707	0.22	ctx-lh-rostralanteriorcingulate
1027	66709	1.20	ctx-lh-rostralmiddlefrontal
1028	66709	1.84	ctx-lh-superiorfrontal
1029	66709	1.08	ctx-lh-superiorparietal
1030	66709	1.00	ctx-lh-superiortemporal
1031	66709	0.91	ctx-lh-supramarginal
1032	66709	0.08	ctx-lh-frontalpole
1033	66709	0.22	ctx-lh-temporalpole
1034	66709	0.10	ctx-lh-transversetemporal
1035	66709	0.57	ctx-lh-insula
2001	66709	0.17	ctx-rh-bankssts
2002	66708	0.16	ctx-rh-caudalanteriorcingulate
2003	66709	0.48	ctx-rh-caudalmiddlefrontal
2005	66709	0.27	ctx-rh-cuneus
2006	66709	0.16	ctx-rh-entorhinal
2007	66709	0.78	ctx-rh-fusiform
2008	66709	1.16	ctx-rh-inferiorparietal
2009	66709	0.90	ctx-rh-inferiortemporal
2010	66707	0.20	ctx-rh-isthmuscingulate
2011	66709	1.07	ctx-rh-lateraloccipital
2012	66709	0.61	ctx-rh-lateralorbitofrontal
2013	66708	0.57	ctx-rh-lingual
2014	66709	0.47	ctx-rh-medialorbitofrontal
2015	66709	0.98	ctx-rh-middletemporal
2016	66706	0.16	ctx-rh-parahippocampal
2017	66709	0.32	ctx-rh-paracentral
2018	66709	0.31	ctx-rh-parsopercularis
2019	66709	0.23	ctx-rh-parsorbitalis
2020	66709	0.33	ctx-rh-parstriangularis
2021	66707	0.18	ctx-rh-pericalcarine
2022	66709	0.75	ctx-rh-postcentral
2023	66709	0.25	ctx-rh-posteriorcingulate
2024	66709	1.07	ctx-rh-precentral
2025	66709	0.80	ctx-rh-precuneus
2026	66709	0.16	ctx-rh-rostralanteriorcingulate
2027	66709	1.24	ctx-rh-rostralmiddlefrontal
2028	66709	1.76	ctx-rh-superiorfrontal

(continued on next page)

Region	Number of MRIs	Percentage Brain Pixels	Label
2029	66709	1.05	ctx-rh-superiorparietal
2030	66709	0.95	ctx-rh-superiortemporal
2031	66709	0.82	ctx-rh-supramarginal
2032	66709	0.10	ctx-rh-frontalpole
2033	66709	0.23	ctx-rh-temporalpole
2034	66709	0.07	ctx-rh-transversetemporal
2035	66709	0.56	ctx-rh-insula
80	2062	0.00	non-WM-hypointensities
72	828	0.00	5th-Ventricle
29	59	0.00	Left-undetermined

Table A4: Brain region segmentation statistics across all labeled regions before pre-processing.

Normalized Label	Percentage Brain Pixels	Region
Accumbens-area	0.08	[26, 58]
Amygdala	0.28	[18, 54]
Brain-Stem	1.88	[16]
CC	0.33	[251, 252, 253, 254, 255]
Caudate	0.59	[11, 50]
Cerebellum-Cortex	9.43	[8, 47]
Cerebellum-White-Matter	2.52	[7, 46]
Cerebral-White-Matter	39.43	[2, 41]
Hippocampus	0.72	[17, 53]
Lateral-Ventricle	2.33	[4, 43]
Pallidum	0.34	[13, 52]
Putamen	0.79	[12, 51]
Thalamus-Proper	1.23	[10, 49]
WM-hypointensities	0.21	[77]
ctx-entorhinal	0.33	[1006, 2006]
ctx-fusiform	1.58	[1007, 2007]
ctx-inferiorparietal	2.10	[1008, 2008]
ctx-inferiortemporal	1.81	[1009, 2009]
ctx-insula	1.13	[1035, 2035]
ctx-lateraloccipital	2.09	[1011, 2011]
ctx-lateralorbitofrontal	1.22	[1012, 2012]
ctx-lingual	1.12	[1013, 2013]
ctx-medialorbitofrontal	0.90	[1014, 2014]
ctx-middletemporal	1.86	[1015, 2015]
ctx-parahippocampal	0.34	[1016, 2016]
ctx-parsopercularis	0.67	[1018, 2018]
ctx-parsorbitalis	0.42	[1019, 2019]
ctx-parstriangularis	0.62	[1020, 2020]
ctx-postcentral	1.53	[1022, 2022]
ctx-posteriorcingulate	0.50	[1023, 2023]

(continued on next page)

**Normalized Label**	**Percentage Brain Pixels**	**Region**
ctx-precentral	2.17	[1024, 2024]
ctx-precuneus	1.58	[1025, 2025]
ctx-rostralanteriorcingulate	0.37	[1026, 2026]
ctx-rostralmiddlefrontal	2.44	[1027, 2027]
ctx-superiorfrontal	3.60	[1028, 2028]
ctx-superiorparietal	2.13	[1029, 2029]
ctx-superiortemporal	1.94	[1030, 2030]
ctx-supramarginal	1.73	[1031, 2031]
ctx-temporalpole	0.45	[1033, 2033]

Table A5: Grouped brain region segmentation statistics after preprocessing.

Appendix B Preliminary Analyses

In this section of the Appendix, we report the preliminary analyses used to select the anatomical model, label encoding, and supervised baseline architecture. For all preliminary analyses, model selection was performed exclusively using a validation subset comprising 10% of the ADNI training split. Results on external cohorts are also reported for completeness, but were not used for model selection. Validation results used for model selection are underlined in the corresponding tables.

B.1 Preliminary Analysis of Linear Model and Nonlinear Model Using Anatomical Features

We extracted 178 FreeSurfer-derived anatomical features from the `recon-all` pipeline, spanning regional brain volumes, cortical surface areas, and cortical thickness values. The complete list of features is reported in Table A3. To verify whether more complex tabular models offered an advantage over logistic regression, we performed an additional comparison between linear and non-linear models using dementia detection as an example task.

Hyperparameters were selected using 5-fold cross-validation. For the linear model, we evaluated both no regularization and L2 regularization with coefficient 1, consistent with the default setting in scikit-learn logistic regression. For the non-linear model, we used a random forest with `n_estimators` in {10, 50, 100}, `max_depth` in {5, 10, 20}, and `criterion` in {`gini`, `entropy`}. To assess whether the comparison depended on sample size, we repeated the experiment using 10%, 30%, and 100% of the training data. As shown in Table B6, performance differences were generally small, which supports the use of logistic regression as the main anatomical baseline.

Dataset	Linear 10%	Non-Linear 10%	Linear 30%	Non-Linear 30%	Linear 100%	Non-Linear 100%
ADNI	0.6882<br>[0.6566, 0.7159]	0.7055<br>[0.6719, 0.7395]	0.7470<br>[0.7165, 0.7798]	0.7398<br>[0.7144, 0.7721]	0.7586<br>[0.7279, 0.7874]	0.7529<br>[0.7203, 0.7834]
NACC	0.7221<br>[0.6723, 0.7693]	0.7510<br>[0.7187, 0.7901]	0.7931<br>[0.7527, 0.8463]	0.8061<br>[0.7546, 0.8516]	0.7954<br>[0.7566, 0.8531]	0.8216<br>[0.7741, 0.8661]
AIBL	0.7111<br>[0.6710, 0.7526]	0.7582<br>[0.7162, 0.8010]	0.8004<br>[0.7576, 0.8468]	0.7933<br>[0.7421, 0.8424]	0.8105<br>[0.7682, 0.8522]	0.8261<br>[0.7847, 0.8651]
OASIS1	0.8328<br>[0.7867, 0.8751]	0.7967<br>[0.7421, 0.8356]	0.8168<br>[0.7673, 0.8538]	0.8150<br>[0.7637, 0.8671]	0.8066<br>[0.7590, 0.8486]	0.8245<br>[0.7706, 0.8772]
OASIS2	0.6796<br>[0.6234, 0.7394]	0.6825<br>[0.6219, 0.7313]	0.7085<br>[0.6393, 0.7522]	0.7038<br>[0.6429, 0.7598]	0.7378<br>[0.6778, 0.7867]	0.7710<br>[0.7180, 0.8173]
OASIS3	0.6621<br>[0.5885, 0.7348]	0.6847<br>[0.6018, 0.7619]	0.7124<br>[0.6396, 0.7831]	0.7009<br>[0.6135, 0.7890]	0.7258<br>[0.6488, 0.7949]	0.7192<br>[0.6310, 0.7955]
OASIS4	0.6326<br>[0.5346, 0.7616]	0.6291<br>[0.5173, 0.7602]	0.6301<br>[0.5293, 0.7618]	0.6376<br>[0.5155, 0.7541]	0.6250<br>[0.5130, 0.7517]	0.6330<br>[0.5103, 0.7514]
MIRIAD	0.9708<br>[0.9584, 0.9825]	0.9799<br>[0.9678, 0.9890]	0.9683<br>[0.9542, 0.9806]	0.9771<br>[0.9687, 0.9856]	0.9733<br>[0.9602, 0.9847]	0.9855<br>[0.9784, 0.9912]

Table B6 Model comparison on dementia detection task with anatomical features. External cohort performance (95% CI) per dataset is also included.

B.2 Preliminary Analysis of Label Encoding

Because dementia progression is not a strictly discrete process, we evaluated different label encodings for the dementia task. In the *soft* encoding, labels were assigned as CN = 0, MCI = 0.5, and AD = 1, and models were trained using log loss. In the *rigid* encoding, both MCI and AD were treated as positive cases, giving CN = 0 and MCI/AD = 1. We also evaluated a standard three-class encoding with labels CN = 0, MCI = 1, and AD = 2.

Preliminary experiments with a linear model using anatomical features showed that the soft-label formulation achieved the best performance on ADNI as shown in (Table B7). For this reason, we adopted the soft-label formulation in the main dementia experiments.

**Dataset**	**Label Encoding**	**AD vs CN**	**AD vs MCI**	**MCI vs CN**	**MACRO**
ADNI	Soft	0.8873<br>[0.8551, 0.9201]	0.7445<br>[0.7099, 0.7742]	0.6926<br>[0.6532, 0.7389]	0.7586<br>[0.7279, 0.7874]
ADNI	Rigid	0.8442<br>[0.8165, 0.8762]	0.7184<br>[0.6887, 0.7412]	0.6565<br>[0.6092, 0.6997]	0.7397<br>[0.7088, 0.7703]
ADNI	Multi-class	0.7107<br>[0.6786, 0.7395]	0.6284<br>[0.5972, 0.6586]	0.5927<br>[0.5503, 0.6425]	0.6439<br>[0.6134, 0.6736]
NACC	Soft	0.9287<br>[0.8977, 0.9532]	0.8588<br>[0.8239, 0.8913]	0.7850<br>[0.7421, 0.8231]	0.8575<br>[0.8301, 0.8842]
NACC	Rigid	0.9157<br>[0.8853, 0.9419]	0.7870<br>[0.7538, 0.8140]	0.7085<br>[0.6719, 0.7385]	0.8037<br>[0.7696, 0.8323]
NACC	Multi-class	0.8215<br>[0.7913, 0.8563]	0.6824<br>[0.6499, 0.7102]	0.6596<br>[0.6172, 0.7014]	0.7212<br>[0.6941, 0.7541]

Table B7 Comparison of label encoding methods with a linear model using brain anatomical features.

B.3 Preliminary Analysis of Baseline Neural Network Model Choice

We performed a preliminary analysis to select a reasonably strong supervised CNN baseline for comparison with the transformer-based models. The analysis was carried out on the ADNI dementia detection task and compared ResNet architectures with different input sizes. Specifically, we evaluated ResNet-18 with input sizes of $(128, 128, 128)$ and $(160, 160, 160)$, and ResNet-50 with input size $(128, 128, 128)$.

As shown in Table B8, ResNet-18 with input size $(128, 128, 128)$ achieved the best overall performance and was therefore adopted as the CNN baseline in the main experiments.

Model Type	Input size	AD vs CN	AD vs MCI	MCI vs CN	MACRO
**ResNet 18**	(128, 128, 128)	0.8674<br>[0.8328, 0.9011]	0.7268<br>[0.6885, 0.7734]	0.6852<br>[0.6449, 0.7286]	0.7598<br>[0.7277, 0.7889]
**ResNet 18**	(160, 160, 160)	0.8106<br>[0.7792, 0.8421]	0.6737<br>[0.6352, 0.7178]	0.6550<br>[0.6082, 0.7002]	0.7141<br>[0.6839, 0.7466]
**ResNet 50**	(128, 128, 128)	0.8382<br>[0.8014, 0.8671]	0.7029<br>[0.6711, 0.7401]	0.6638<br>[0.6229, 0.7073]	0.7350<br>[0.7012, 0.7653]

Table B8 Comparison of model performance with different input sizes and model complexity. We adopt the best performing model as the baseline supervised model.

Appendix C Additional Model and Training Details

C.1 Additional details on MAE pretraining and downstream adaptation

The pretraining cohort consisted of UKBB, HCP, CamCAN, and IXI. Ten percent of UKBB participants were excluded from MAE and ASP optimization and used for linear-probe monitoring during pretraining. This participant-level holdout, comprising 6,950 MRI scans, was subsequently used as the internal held-out cohort for age estimation.

Following pretraining, the encoder was adapted using the task-specific strategies described in the main Methods: linear probing for Parkinson's disease and IDH mutation prediction, and LoRA for the remaining downstream tasks. Full fine-tuning was evaluated as an ablation against LoRA. For regression tasks, we used a one-dimensional output and optimized the model with mean squared error (MSE) loss. For classification tasks, we used a two-dimensional output and optimized with binary cross-entropy.

For healthy aging, we finetuned the model on healthy subjects from the pretraining cohort and evaluated generalization on healthy subjects from ADNI and NACC. For dementia diagnosis, we finetuned on ADNI and evaluated on a held-out ADNI test cohort as well as external cohorts from NACC, AIBL, OASIS1-4, and MIRIAD. For Parkinson's disease diagnosis, tumor IDH mutation detection, and epilepsy diagnosis,

we used task-specific train/test splits from PPMI, UCSF-PDGM, and OpenNeuro, respectively.

C.2 Additional details on ASP architecture

The ASP decoder was designed to make the ViT embedding carry anatomical information, rather than allowing a large segmentation head to solve the task on its own. For this reason, we avoid skip connections and use a small bottleneck decoder that receives the global image embedding as input. This differs from standard U-Net-style segmentation models, where local features from early layers are repeatedly fused into the decoder.

To handle multiple anatomical regions efficiently, the decoder is conditioned on a learned region embedding. At each training step, one anatomical region is selected and the model predicts a binary mask for that region. This avoids producing dense logits for all 39 regions at once, which would be memory intensive for full 3D volumes.

The decoder starts from a 768-dimensional image embedding summed with the selected region embedding. This representation is then passed through six 3D transposed-convolution blocks, which upsample the feature map from 1^3 to 128^3. Each block is followed by BatchNorm and ReLU, except for the final layer. The full decoder contains only around 15 million parameters, which is much smaller than a typical 3D U-Net. This keeps the segmentation head lightweight and encourages the ViT encoder to learn the anatomical semantics instead of delegating the task to the decoder.

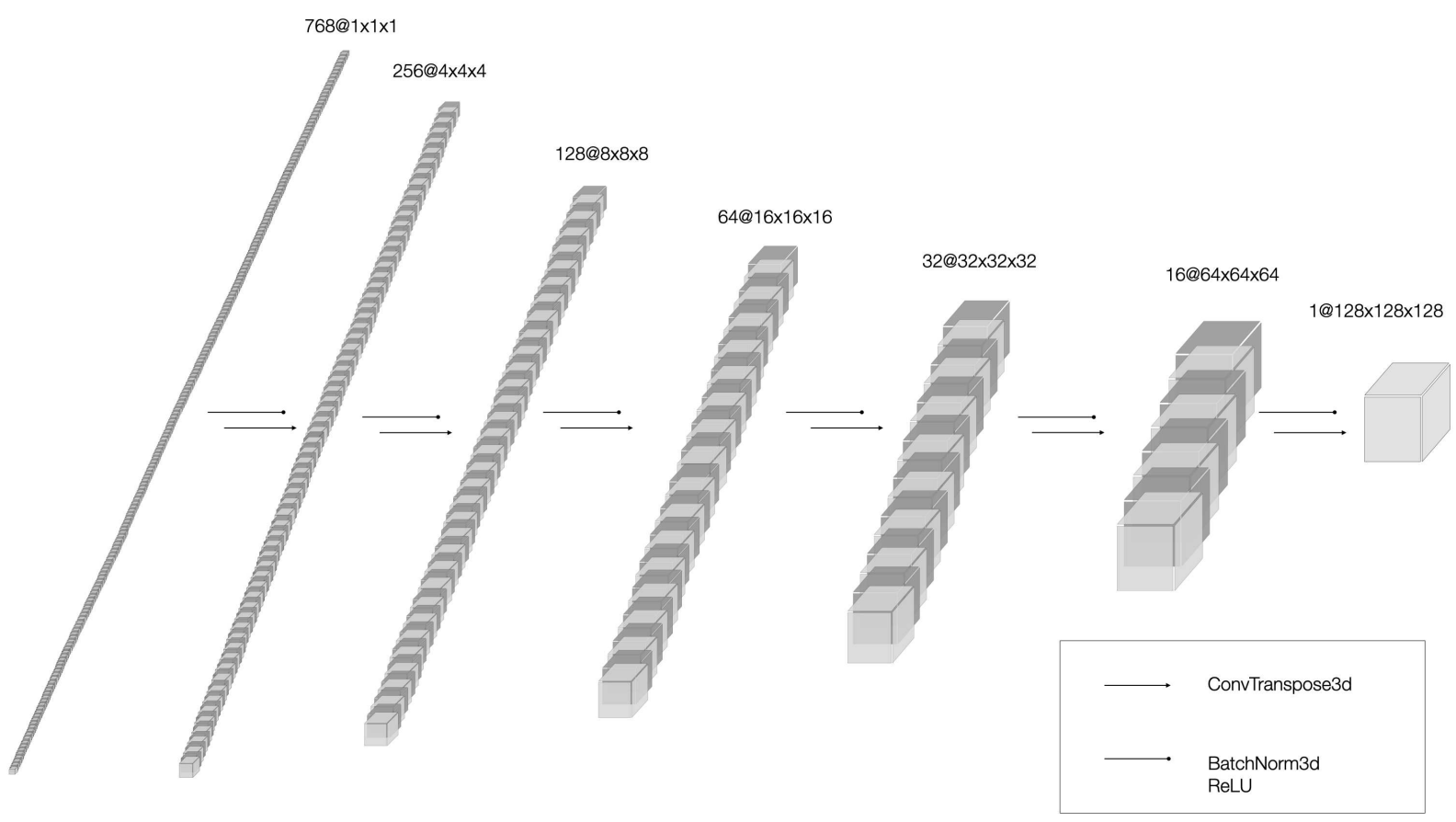

Fig. C1 Lightweight region-conditioned 3D mask decoder used for ASP.

C.3 Ablation Study on ASP Training

We performed an ablation study to evaluate the main training choices used in ASP. For Alzheimer's disease and epilepsy detection, we compared full fine-tuning (FF) with LoRA. As shown in Table C9, both methods performed similarly for Alzheimer's disease, while LoRA improved epilepsy detection. For age estimation, we additionally evaluated several ASP training ablations, including removing MAE alternation, encoder freezing, MAE-only pretraining, and training from scratch. The results in Table C10 correspond to the variants shown in Figure 4(e). We evaluate age estimation on UK Biobank as the internal test cohort and NACC as the external test cohort. To avoid confusion with masked autoencoding, we report mean absolute error as "absolute error" in the table.

The full ASP model achieves the best performance on both cohorts. Removing alternating masked-autoencoder reconstruction updates reduces performance, and removing both alternating updates and the initial encoder-freezing stage reduces it further. The masked-autoencoder-only model remains a strong baseline, but it is outperformed by the ASP variants that add anatomical supervision. Training from scratch performs worst, showing that ASP benefits from starting from a pretrained masked autoencoder.

Task	Evaluation Setting	ASP (FF)	ASP (LoRA)
AD (Train: ADNI)	ADNI	0.7800	**0.7815**
	NACC	0.8260	**0.8264**
	AIBL	**0.8318**	0.8232
	OASIS1	**0.8733**	0.8083
	OASIS2	0.8092	**0.8439**
	OASIS3	**0.7454**	0.7376
	OASIS4	**0.6622**	0.6457
	MIRIAD	0.9815	**0.9866**
AD (Train: NACC)	ADNI	**0.7676**	0.7665
	NACC	0.8438	**0.8570**
	AIBL	**0.8314**	0.8216
	OASIS1	0.9091	**0.9120**
	OASIS2	0.8105	**0.8308**
	OASIS3	**0.7748**	0.7658
	OASIS4	**0.6658**	0.6502
	MIRIAD	**0.9942**	0.9914
Epilepsy Detection	Internal	0.6398	**0.7507**
	External	0.8359	**0.8716**

Table C9 Comparison of ASP (FF/Full-Finetuning) and ASP (LoRA) AUROC performance across classification tasks.

Model	Internal: UK Biobank			External: NACC		
	**Pearson $r\uparrow$**	**MSE $\downarrow$**	**Abs. error $\downarrow$**	**Pearson $r\uparrow$**	**MSE $\downarrow$**	**Abs. error $\downarrow$**
**ASP (LoRA)**	**0.9361**	**10.38**	**2.54**	**0.8589**	**29.26**	**4.19**
ASP (Full-Finetuning)	0.9333	10.83	2.60	0.8464	32.06	4.32
No masked-autoencoder alternation	0.9291	11.49	2.67	0.8324	34.51	4.46
No alternation and no freezing	0.9220	12.59	2.78	0.7987	40.50	4.71
Masked autoencoder only	0.9159	13.61	2.89	0.8043	45.35	5.20
From scratch	0.8736	19.88	3.51	0.7407	55.84	5.79

Table C10 ASP training ablation evaluated on age estimation. Absolute error denotes mean absolute error in years.
